# 

**Published:** 1886-01

**Authors:** 


Whole Number, CCLXXX.

### JANUARY, 1886.

Number 6. Volume XXV.

LIBRARY INDEXED S.G.O.

### The Buffalo Medical and Surgical Journal

ESTABLISHED IN YEAR 1844.

INDEX MEDICUS

**EDITORS:**

**THOS. LOTHROP, M. D.** **A. R. DAVIDSON, M. D.**

ASSOCIATE EDITORS:

FLOYD S. CREGO, M. D. CARLTON C. FREDERICK, M. D.

FRED. R. CAMPBELL, M. D. HERBERT MICKLE, M. D.

Entered at the Post-Office at Buffalo, N. Y., as Second-class Mail Matter.

